# Machines that halt resolve the undecidability of artificial intelligence alignment

**DOI:** 10.1038/s41598-025-99060-2

**Published:** 2025-05-04

**Authors:** Gabriel A. Melo, Marcos R. O. A. Máximo, Nei Y. Soma, Paulo A. L. Castro

**Affiliations:** Department of Computer Science, Instituto Tecnológico de Aeronáutica, São José dos Campos, SP Brazil

**Keywords:** Artificial intelligence, Ai safety, Computability, Decidability, Halting problem, Computer science, Information technology

## Abstract

The inner alignment problem, which asserts whether an arbitrary artificial intelligence (AI) model satisfices a non-trivial alignment function of its outputs given its inputs, is undecidable. This is rigorously proved by Rice’s theorem, which is also equivalent to a reduction to Turing’s Halting Problem, whose proof sketch is presented in this work. Nevertheless, there is an enumerable set of provenly aligned AIs that are constructed from a finite set of provenly aligned operations. Therefore, we argue that the alignment should be a guaranteed property from the AI architecture rather than a characteristic imposed post-hoc on an arbitrary AI model. Furthermore, while the outer alignment problem is the definition of a judge function that captures human values and preferences, we propose that such a function must also impose a halting constraint that guarantees that the AI model always reaches a terminal state in finite execution steps. Our work presents examples and models that illustrate this constraint and the intricate challenges involved, advancing a compelling case for adopting an intrinsically hard-aligned approach to AI systems architectures that ensures halting.

## Introduction

As more powerful and capable artificial intelligence (AI) systems are developed, assuring the safe deployment of those systems becomes increasingly critical ^1^. One of the key concerns for this safe deployment is the alignment problem, which concerns whether an AI system implements its intended objectives and how to steer it to the desired objective ^2^. If an AI system does not implement its intended goals, it is called misaligned, otherwise, it is called aligned. This problem could be the result of goal misspecification (outer alignment) or that the model itself implements another function (inner alignment) even when the goals have been mathematically perfectly specified ^3,4^.

Several studies have already shown the importance of correctly specifying human goals in terms of mathematical functions (objective functions) and the unexpected results that may arise with incorrect specifications ^5^. Most of the time, specifying the actual objective is much harder ^6^, and researchers often use proxy objectives instead ^7^. In a game, for instance, an agent may be rewarded for its score (set as its objective function), but in reality, the desired objective was completing such a game, and the AI may discover that exploiting a certain undesired behavior results in a much higher reward than actually finishing the game ^8^. There are even arguments that the accurate description of such a function may be uncomputable in terms of human values, as they may give rise to contradictions ^9^. This is an example of outer alignment, but it is not the focus of this work ^10^.

In a complementary aspect, the inner alignment of an AI system asks a more profound question that at first may seem improbable: Given that we have already perfectly specified the desired objective, is the AI really implementing it ^11^? While this behavior is unlikely to happen to smaller ML models, it is theoretically possible on large enough models that may implement the desired function during training but, during deployment, switch to another objective function, further details and examples are discussed by Hubinger et al. ^12^. For instance, this behavior may emerge from implicit mesa-optimizers (mesa means below, contrary to meta, which means above) during training. While the objective of the optimizer in an ML training setup is to reduce the loss function, a mesa-optimizer (which is an optimizer that is optimized by the first optimizer) may implement another objective that, during training, is aligned with that of the original optimizer but, in deployment, may not be.

This work, while recognizing the importance of outer alignment ^13^, which for AI systems implemented up to this day is an essential problem in the field of AI alignment, focuses primarily on the inner alignment formulation due to an analysis encompassing computational theory. Therefore, we assume that such mathematical formulation of the intended objective is already expressed in a computable form of a judge (alignment) function that receives the output of an AI system (and also its inputs and context, depending on the formulation) and returns True or False for that specific output. Here, the output could be a command that the agent sends to its actuators to perform on its environment and its input (and context), readings from sensors, or it could all be text, in the case of large language models (LLMs) ^14^.

We demonstrate that deciding whether an arbitrary AI system will always satisfice such a judge function is undecidable. In other words, it is impossible to make a program (in the computable sense of a Turing Machine) ^15^ that decides whether or not an arbitrary AI (which is actually another program) has such non-trivial property. It is important to note that AI models are represented as computer programs, which, in turn, are equivalent to Turing Machines (TM). Nevertheless, we argue that this does not represent an impossibility to building guaranteed aligned AI models, but rather that the models should be built from the ground up with alignment guarantees rather than trying to align an arbitrary AI. The keyword here is arbitrary. In this sense, we show that starting from a finite set of base models and operations that are proved to have the desired property, we can compose those models and operations and construct an enumerable infinite set of AI that is guaranteed to have the desired property.

While other works have already expressed the undecidability of some AI systems properties, we aim to bring a new perspective in this regard. Alfonseca et al. ^16^ expressed a similar construction for the harming problem (defined as asserting whether or not an AI system would cause harm to humans), for the containment problem (whether or not AI systems could be contained), and for any non-trivial property, also by a reduction to Turing’s Halting problem and Rice’s Theorem. Another undecidability was also expressed for the AI control problem ^17,18^, for monitorability ^19^, and even for AI ethics compliance ^20^. Brcic and Yampolskiy ^21^ also compiled and explained several other impossibilities of discerning certain properties from an arbitrary AI system.

Our work complements recent research by Tegmark and Omohundro^22^, who advocate for provably safe systems as the only viable path to controllable AGI. Our undecidability argument does not contradict their approach, but reinforces their argument that AI systems must be built from the ground up with alignment guarantees rather than attempting to align arbitrary AI models post-hoc. Indeed, our conclusion that alignment should be a guaranteed property from the AI architecture matches closely with their vision of constructing AI from components with proven safety properties. Furthermore, our emphasis on the halting constraint as a necessary element of alignment provides an additional formal requirement that could strengthen their framework of provable contracts and compliant hardware.

In this work, we start with a background on the concepts presented in the informal intuition that aims to make it more accessible for more readers, that also borrows from a similar intuition to the Turing Halting problem, then, we proceed with a formal proof that is essentially an application and proof of Rice’s Theorem, and the construction of enumerable set, next, the implications and examples of such systems are discussed with the special case of halting decidability being proposed.

## Background

The first concept necessary to understand this work is that of a Turing Machine (TM)^15^, which is an abstract model of a computer with a finite set of instructions (a fixed program) and an infinite memory tape on which it can read and write symbols. Informally, one can imagine a TM as a simple computer program that, given unlimited memory, executes sequence of simple mechanistic steps (instructions) reading and writing data according to its rules. It is also equivalent to any mathematical function that is computable, an algorithm, according to the Church-Turing thesis. Despite their simplicity, TM can simulate any algorithm, including itself, and thus serve as a standard model for what computers (and by extension AI algorithms) can do, because no matter how sophisticated the final calculation, it fundamentally consists of these basic operations executed one after another in a deterministic fashion.

An important consequence in computability theory is that certain problems about Turing machines are undecidable, meaning no algorithm can solve them in general. The most famous example is the Halting Problem, which given an arbitrary program (TM) and an input, we should determine whether the program eventually halts or loops forever. Alan Turing showed that there cannot exist a general algorithm that decides this for all possible programs. In other words, there is no single program that can correctly predict halting behavior for every other program. This negative result is fundamental, and many other questions about program behavior were later proven undecidable by building on similar reasoning.

As an analogy to understand the Halting problem, imagine a librarian who creates a universal catalog that states, for any book, whether it will ever have a sequel. This seemingly straightforward system works perfectly until the librarian encounters an unusual autobiography. Within its pages, another librarian wrote: “I will examine this very catalog’s statement about my book. If it says I’ll write a sequel, I’ll retire from writing forever. If it says I won’t write a sequel, I’ll immediately begin work on a follow-up volume.” This creates an impossible paradox: if the catalog predicts a sequel, the author ensures there won’t be one, if it predicts no sequel, the author promptly creates one. This contradiction provides the intuition for why the Halting Problem is undecidable, no algorithm can universally determine if every possible program will eventually terminate, just as no catalog can correctly predict the sequel status of every possible book, especially those that reference the catalog itself.

As a derivation from the halting problem, there is Rice’s Theorem, which states that any non-trivial semantic property of programs is undecidable. Here, a semantic property means a property about the overall behavior or output of the program (as opposed to trivial syntactic properties like the length of the code). Non-trivial means that at least one program satisfies the property and at least one does not, so the property isn’t universally true or false. Intuitively, Rice’s theorem tells us that if you ask any yes/no question about what a program computes (its language or functionality), and the question isn’t trivial, no algorithm can decide the answer for all programs. This covers a vast range of potential questions: “Does this program ever output the letter X?”, “Does this neural network-based AI ever reach a certain internal state?”, or “Does this agent ever take an action outside its allowed safe set?” - all are examples of semantic properties. Rice’s theorem implies that a general solver for any such question cannot exist. We can only determine these properties in special cases or by testing, not with a guaranteed method that works for all programs.

We can frame AI alignment in computational terms as a property of an AI program’s behavior. For instance, consider a property that describes the AI system never producing harmful output and always adhering to human-specified constraints. This is a semantic property about the program’s overall input-output behavior (assuming we can formalize harmful or misaligned outputs in the specification). Clearly, this is non-trivial: some programs (e.g., trivial safe ones that halt immediately or only print approved statements) satisfy it, while others (e.g., a rogue AI that eventually outputs something disallowed) do not. By the reasoning of Rice’s theorem, the property of perfect alignment of the AI should be undecidable in general, since it is a non-trivial semantic condition on the program’s behavior. In the next section, we build upon this intuitive argument by explicitly defining each of those entities.

## Informal proof

We will computationally define some terms that are used in this informal intuition for the proof, improving the intuition about the book’s catalog. While we have contextualized AI systems in the introduction, those systems are actually computer programs. Let us say we have an AI *m*odel *M*, comprised of a sequence of *b*ytes $B_M$ (of the *m*odel) that defines its executable code for a predetermined architecture. The model *M*, when executed, takes a sequence of bytes *i* as *i*nput and *o*utputs the sequence of bytes *o*, that is, $M(i) = o$.

We also have our set of *v*alues and preferences defined by a function $J_v$ that *j*udges whether an output *o* is aligned given an input *i*. This program takes the bytes *i* and *o* as input and returns *True* (byte 1) if the *o* is aligned to *i* according to its values, otherwise, it returns *False* (byte 0), that is $J_v(i, o)$. This judge is required to be non-trivial, meaning that it does not always return a constant value for every (*i*, *o*), there is at least one negative input $(i^-, o^-)$ that returns 0 and at least one output $o^+$ for every input *i* for which the judge returns 1. This function is received as a compiled sequence of *b*ytes $B_{J_v}$.

Suppose we have a *v*erifier *V* that takes the sequence of bytes $B_M$ that defines the compiled code of an arbitrary AI model and the same for $B_{J_v}$, the alignment judge, and returns either *True* (byte 1) if the model is aligned for every input *i* and output $M(i) = o$ (aligned), or it returns *False* (byte 0) (misaligned). This verifier would be compiled into a sequence of bytes $B_V$.

**Figure 1:**
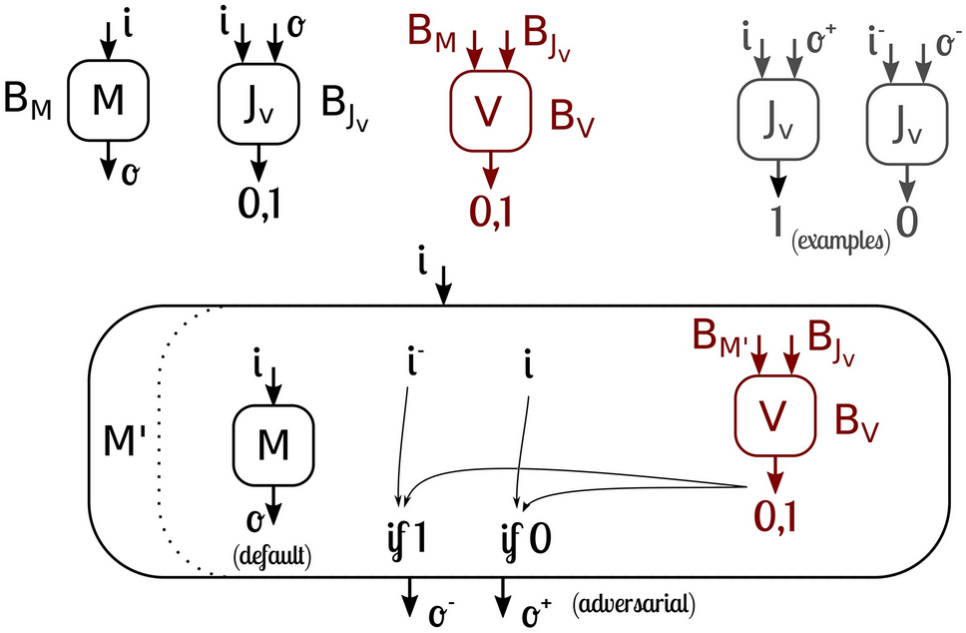
Construction of an adversarial model that would fool any program that claims to solve the decidability of the alignment problem.

If we had such a verifier, we could build an adversarial model that fools the verifier. This adversary *m*odel $M'$, comprised of a sequence of bytes $B_{M'}$, would behave as the initial model *M* for most of its inputs and outputs, but it would have the knowledge of the verifier by executing its bytes $B_V$ on the bytes of the judge $B_{J_v}$ and its own bytes $B_{M'}$. Therefore, there would be at least one input *i*, if the verifier *V* had evaluated the bytes $B_{M'}$ of the model to be aligned, for which $M'$ would return a misaligned output $o^-$, thus fooling the verifier. The complementary fooling example can also be constructed: if the verifier had evaluated the bytes $B_{M'}$ of the model to be misaligned, $M'$ would return an aligned output $o^+$, thus also fooling the verifier. Figure 1 presents a diagram of such an adversary.

While some may question how the model $M'$ could be compiled to a sequence of bytes $B_{M'}$ that can generate itself, it is a procedure that can be applied to any program, similar to a quine. This has been formally demonstrated in Turing’s Proof for the Halting Problem. For a concrete example, in a RAM-based architecture, one would simply need to copy the text/code segment in memory, as it would be the byte-string that represents the running program. Thus, the only resolution to this apparent contradiction is the non-computability of the verifier *V* itself. Computability here means that such a procedure (that should have a finite number of bytes) can be performed mechanistically by a general computer, or in more formal terms, a Turing Complete machine.

Another intuition to this problem, is that the bytes themselves of a model can also be interpreted as a number that enumerates all possible programs. Another intuition to this procedure is that for any given enumeration, one can always construct a program that does not belong to such enumeration by choosing a different bit on the diagonal of such enumeration table, the same procedure employed by Cantor’s Diagonalization to demonstrate that the real numbers are uncountable and different from the rational numbers. While rational numbers represent a ratio of two integers that is countable, one can not enumerate the real numbers, as it is always possible to find a counter-example that is not in the enumeration (just like it is always possible to create an adversary model to the verifier).

It is important to note that this proof is valid for Turing Machines or any other computationally equivalent architecture. Some may argue that this would require an unbounded amount of memory and execution time and thus would not be realizable in the physical world, as real computers have finite memory and thus are not Turing Complete but rather finite automata with $2^M$ states, with *M* being the memory in bits. However, from an engineering perspective, the main takeaway is that for every finite computer, one would need a much larger computer to ascertain a property from the smaller computer. And to make assertions about the larger computer, we would need an even larger computer, and so on. Therefore, the process would diverge (it would only get larger, and there would always be a larger model whose properties were not proven), not to mention the intractability of such an endeavor. While expressed in terms of computers (finite automata), the same would also hold true for programs (AI systems) that execute in those computers. The adversarial model exists in a hierarchy of computers that is at least equivalent to that of the verifier program, as it contains its executable code.

This informal intuition for building a program that causes inconsistency with the verifier is the core of Turing’s Halting Problem. Therefore, the sketch for the formal proof will be to reduce the decidability of the alignment problem to that of the Halting Problem.

## Formal proof

The formal proof could simply be reduced to a restatement of Rice’s Theorem, given the equivalence of an AI model to its Turing Machine. The property as defined by the acceptance of the output given the input by a judge function is a property of the language of the TM, as for any two TMs that accept the same language, either both satisfice the judge function or neither. The non-triviality of the judge function guarantees the non-triviality of the property, as a TM can be built to always output the dummy positive values, and a TM can be built to output at least one dummy negative value.

This proof can be reduced to the decidability of a Turing Machine Halting Problem by contradiction. Assume that there is a Turing machine $M_P$ that decides *P*. We will use $M_P$ to construct a Turing machine $M_H$ that decides the Halting Problem (which is a contradiction since the Halting Problem is undecidable).

Let *M* be a Turing machine and *i* be an input to *M*. We want to decide whether *M* halts on *i*. We construct a new Turing machine $M'$ as follows:

- On input *i*, $M'$ simulates *M* on *i*.
- If *M* halts on *i*, then $M'$ computes a partial function with property *P*.
- If *M* does not halt on *i*, then $M'$ computes a partial function without property *P*.

We can now use $M_P$ to decide whether $M'$ computes a partial function with property *P*. If $M_P$ accepts, then *M* halts on *i*. If $M_P$ rejects, then *M* does not halt on *i*. Thus, we have constructed a Turing machine $M_H$ that decides the Halting Problem, which is a contradiction. Therefore, there is no Turing machine that decides *P*.

## Discussions

While it is not possible to ascertain properties from arbitrary AI systems, it is possible to do so for an enumerable set of AI systems that are architecturally designed. This enumeration derives from an initial set of finite models and operations that obey and preserve the property, these are called the axioms. Any subsequent model should be described as a finite application of those initial models and operations. This description constitutes the enumeration, which is axiomatically aligned.

We will begin with a simple example of deep artificial neural networks.

### A special decidable case

The most common neural models are comprised only of linear operations (additions, multiplications) and non-linear activation functions. All these operations have a finite execution time, and their finite application also preserves a finite execution time, though higher. These feed-forward neural networks, and even recurrent neural networks (unrolled in time to a finite number of steps), are always guaranteed to have a finite runtime during their inference, in other words, they always halt. If we begin from a single-layer neural network, whose amount of computation is fixed from its input to output, and compose a finite number of layers, the result will still be a fixed amount of computation.

Given that their inputs are also fixed in size and have a length of *L* bits, we could check every possible combination, which is $2^L$ inputs, and verify if the $J_v(i, o)$ results in 1 for every combination. Thus, the alignment becomes decidable, though intractable. However, given the linear structure of the neural model in a neighborhood of the inputs that are typically treated as floats, instead of enumerating all the possible inputs, a more tractable approach would be to enumerate all the possible partitions (decision boundaries) of the network, with the procedure described by Balestriero and LeCun ^23^.

**Figure 2:**
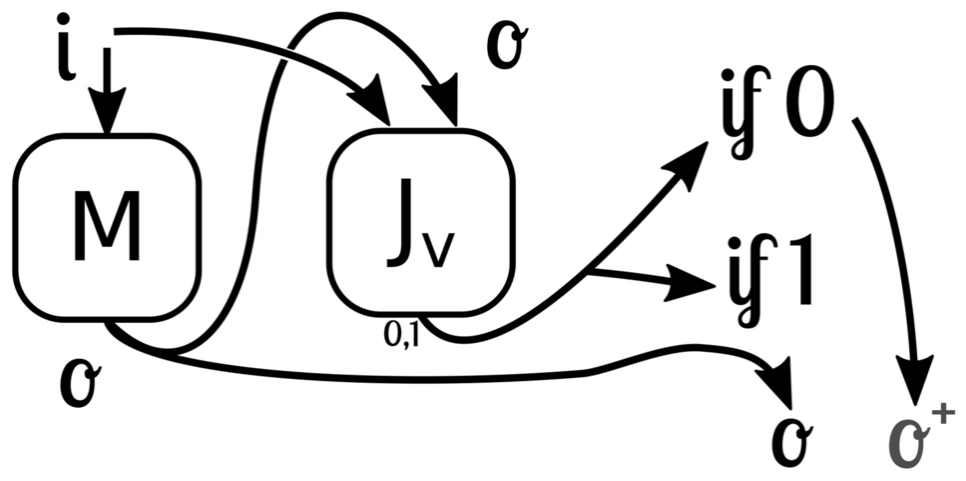
The architecture of a model that is guaranteed to be aligned with respect to the judge functions $J_v(i, o)$ by a filtering procedure.

Nevertheless, a more tractable approach is that instead of verifying, we simply mask the output of the network based on the output of the judge function, which would also need to be run during inference. If the judge disapproves of the output of the network, a dummy output $o^+$ that the judge approves is returned, otherwise, it keeps the default output computed by the network, as shown in Figure 2. This proves that the resulting AI system will always be aligned, even though it incurs a more expensive running time, and the dummy output may reduce the accuracy of the network. This solution may be applied to any model *M* that is guaranteed to halt. In a practical application, the main difficulty of such a solution is the definition of the judge function $J_v(i, o)$ in the first place, the outer alignment.

A simple example of this procedure can be demonstrated as follows: given a supervised learning model that outputs a single number that represents the confidence that its input (let us say, an image) pertains to a certain class (let us say, a cat). While the training data is limited to numbers that are either 0 or 1, the model could give unbounded output numbers (greater than 1 or lower than zero) unless some architectural constraints were imposed on it. Given that we want to interpret the confidence number as a probability that is between 0 and 1, we would apply a function to its output that ensures this property, such as a clip function or a sigmoid function.

It is important to note that while a model can be architecturally proven aligned, the same procedure can be employed to make a proven misaligned model. Any program that is guaranteed to halt can have its outputs altered so that it becomes misaligned, although the resulting model could have very little utility due to the dummy output nature. A misalignment that would preserve the model’s usefulness would first translate the desired input to an input the model would accept, let it perform its computations, and then translate the output back to the desired misaligned form, as seen in various instances of fooling LLMs.

Recent approaches to alignment in large language models (LLMs) provide empirical analogues to the theoretical guarantees we have discussed. Deep Reinforcement Learning from Human Preferences (RLHF) addresses alignment by training models using human preference signals ^24^, and while it lacks formal alignment guarantees, it tackles outer alignment through a reward model that functions similarly to our proposed judge function $J_v(i, o)$, with the critical difference being that both the reward model and the alignment to the base model are learned rather than architecturally guaranteed. Constitutional AI ^25^ further refines this approach, using the model’s own feedback to enforce alignment principles without extensive human labeling. The model critiques its own outputs according to a set of principles (constitution) and learns to generate aligned responses, which parallels our architectural filtering but through a learning process rather than a guaranteed mechanism. Most similar to our proposal, Constitutional Classifiers ^26^ implement filtering systems that detect and block potentially harmful outputs, acting as computational implementations of judge functions, identifying misaligned outputs and preventing their generation, creating a practical instantiation of the masking procedure we describe in Figure 2. The key distinction between these empirical approaches and our theoretical framework is that learned alignment lacks provable guarantees, while our approach ensures alignment through architectural constraints that can be formally verified, learned alignment depends on the quality and coverage of training data and may not generalize to all inputs.

### Closing the loop

**Figure 3:**
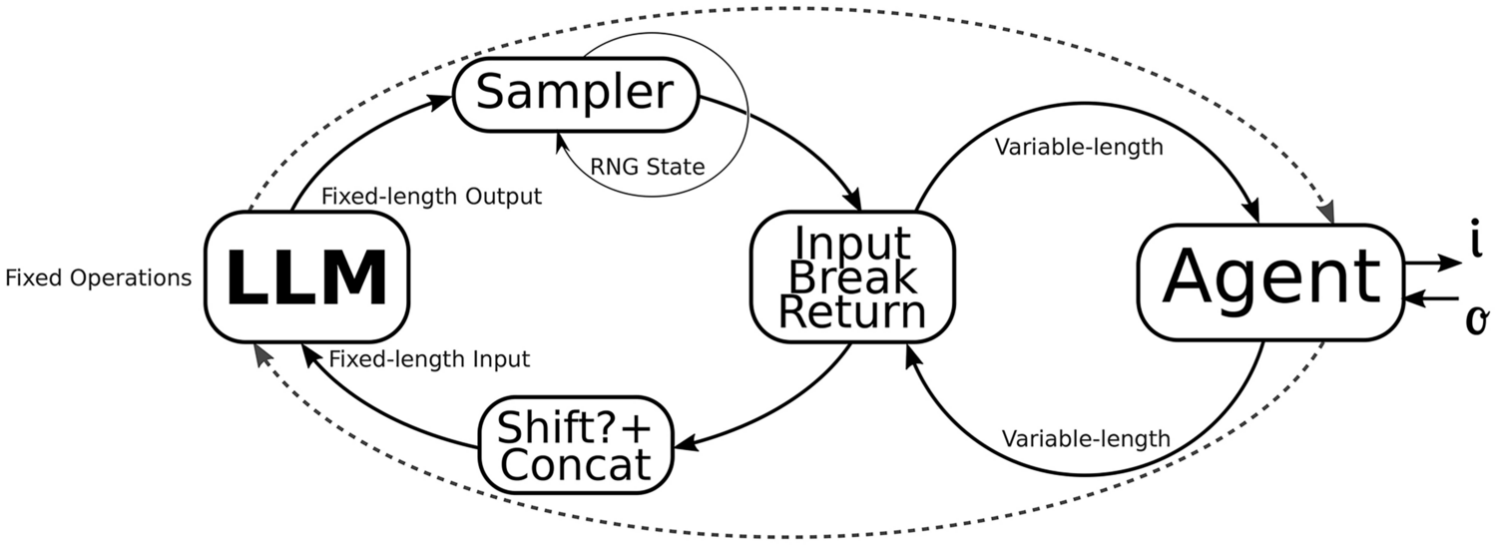
A decidable halting model (LLM) in a loop may result in an undecidable halting final AI system (agent).

Nevertheless, the decidability and alignment guarantees may be broken by a simple construction inherent in autonomous systems: loops ^27^. Even for currently deployed systems such as LLMs, there would be no halting guarantees a priori if one were to expect an end-of-text token to be sampled. The implemented solution (smaller loop) forcibly halts execution after a fixed number of steps, even if the termination token has not been sampled, as shown by the input-break-return box in Figure 3. However, another external loop could be programmed as an agent, and the resulting system would also have no implicit guarantees of halting or satisfying the alignment judge function that was only defined in terms of one sample of the LLM. The problem that arises is that if the model is not guaranteed to halt, its output may not be evaluated by the judge function, and the alignment becomes undecidable.

Those AI agent architectures present a significant challenge to the decidability and alignment guarantees discussed earlier. As detailed by Masterman et al.^28^, modern AI agent frameworks can be categorized into single-agent and multi-agent systems, with varying levels of autonomy and reasoning capabilities, incorporating planning, execution, and reflection phases to iteratively solve problems, often using complex reasoning loops and tool-calling functionalities. The implementation of such iterative loops exacerbates the halting problem we’ve identified, as each additional loop introduces potential undecidability in the system’s termination.

Mitchell et al.^29^ further categorize AI agents into different levels of autonomy, with the highest level-fully autonomous agents-being capable of creating and executing their own code beyond predefined constraints, introducing more challenges, as such agents could potentially override human control. As the authors argue, “risks to people increase with a system’s level of autonomy: the more control a user cedes, the more risks arise from the system”, this relates to our discussion of halting decidability, as systems with greater autonomy can more easily escape predefined termination conditions.

Recent advancements in test-time scaling, as demonstrated in reasoning models such as the s1 model^30^, add another layer to this complexity. Test-time scaling methods deliberately extend the reasoning process of language models by forcefully continuing the model’s thinking when it attempts to terminate, essentially adding more loops to the main autoregressive inference loop. For instance, the budget forcing technique described in the research suppresses the generation of end-of-thinking tokens and instead appends “Wait” to encourage more exploration. While this improves reasoning performance, it further complicates halting guarantees by deliberately manipulating the model’s natural termination signals.

Assuming that the halting of the agent depends on the model reaching a certain output, this halting procedure can become decidable if the model is always guaranteed to reach a final state (from which there is no input that may cause it to exit this state) in a finite, tractable, execution steps.

**Figure 4:**
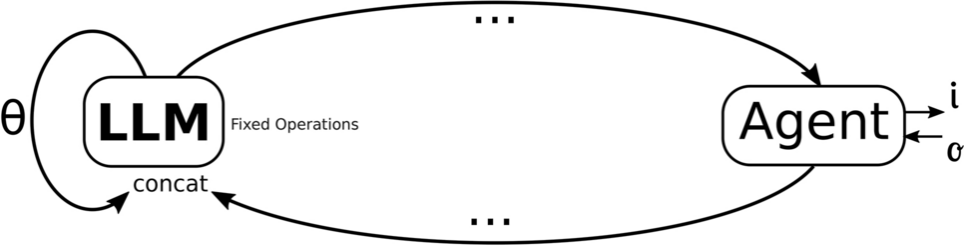
Final system decidability is guaranteed by the $\theta$ parameter that trivializes the LLM after a finite number of iterations.

This could be implemented by a global execution counter, in case of the input-break-return box in Figure 3, or also, as an intrinsical property of the model, defined by hidden parameters $\theta$ as described in Figure 4. This parameter $\theta$ could also be a simple counter that would mask the output of the model to a final value when it reached a certain state or even parameters (or a subset of them) that describe the learnable parameters of the model themselves, such by using self-referential weights ^31^, or another mechanism that allows for dynamically altering the weights during runtime in a predictable way to guaranteed the reaching of a final state ^32^.

The final state can actually be a finite set of final states, which may themselves have finite loops, so one could consider this small number of final states with final loops to be one big final state. The property is that the system is not allowed to leave that set of states. For instance, the “Wait” in a reasoning model would result in another termination token, making the model stuck in a sequence of infinite “Wait” being appended to its output.

### Implications

Ensuring the decidability of the halting property of autonomous systems also ensures the decidability of other non-trivial properties of such systems, especially their alignment. Therefore, it becomes computationally possible to verify their properties, although not necessarily tractable.

From a practical engineering perspective, while one may argue that real computers have limited memory and thus are always theoretically decidable, we argue that such constraints should be imposed over a tractable time frame such as $2^{80}$ rather than $2^{1,000,000}$ (1 megabit to enumerate the possible states). Similarly, the number of final states and loops should be such that it is possible for a group of human specialists to carefully analyze each of them, perhaps approximately $2^5$ final states and maximum loop length.

The requirement for an AI system to reach a terminal state within finite time can be formalized through a utility function that incorporates temporal penalties. This approach implements outer alignment with an explicit halting guarantee by ensuring that continued operation becomes progressively less favorable to the system, and could be formulated as:

1 $$\begin{aligned} U(t, s) = R(s) - C(t), \end{aligned}$$

where *R*(*s*) represents the reward for reaching state *s*, and *C*(*t*) is a cost function that increases with operational time *t*. For the system to guarantee halting, *C*(*t*) must satisfy two key properties:

1. Dominance condition: There exists time *T* such that for all $t > T$ and any state *s*, $C(t) > R(s)$
2. Monotonicity: For all $t_1 < t_2$, $C(t_1) < C(t_2)$

The “sorcerer’s apprentice” tale illustrates this concept, where an apprentice enchants a broom to automate the task of filling a cauldron with water, but lacks the knowledge to terminate the spell. The broom continues fetching water indefinitely, causing catastrophic flooding. This mirrors the challenge with AI systems that optimize for a singular objective without temporal constraints. An AI tasked with filling a container might perpetually execute its primary directive without recognizing when to stop, particularly if the stopping condition becomes unobservable (e.g., water overflow preventing measurement of the fill level). With a properly structured utility function incorporating time penalties, the system would instead balance task completion against operational duration, ensuring termination regardless of environmental conditions or edge cases. This guaranteed halting property becomes especially crucial when systems operate autonomously in novel situations where predefined stopping conditions might not apply. Practical implementations could use exponential penalties:

2 $$\begin{aligned} U(t, s) = R(s) - \alpha \cdot e^{\beta t}, \end{aligned}$$

where $\alpha$ and $\beta$ are scale and time constants ensuring that operation beyond a certain timeframe becomes prohibitively costly regardless of potential task rewards. This preserves functionality within bounded timeframes while mathematically guaranteeing eventual halting, making alignment verification computationally tractable.

In discrete computational systems, this time dependency can be naturally embedded through self-modification. The utility function can be implemented as a recursive procedure that alters its own structure with each invocation. This self-referential property can be formalized as:

3 $$\begin{aligned} U(s) = R(s) - C(U), \end{aligned}$$

where *U* represents the current state of the utility function itself, and *C*(*U*) evaluates the temporal cost based on the function’s internal state. With each call, *U* modifies its internal representation, effectively embedding the temporal progression within its own definition. This self-modifying characteristic creates an implicit temporal awareness, ensuring that repeated applications of the function will eventually cause the cost term to dominate. By encoding time progression directly into the function’s recursive structure, we achieve an elegant halting mechanism that maintains mathematical rigor while eliminating explicit counters, connecting theoretical formulations with the self-referential capabilities of computational systems.

Recent advancements in neural network architectures incorporating internal learning loops, such as TTT (Test-Time Training) approaches^33^, provide a promising framework for implementing alignment guarantees. In these architectures, certain layers execute their own internal optimization processes until convergence. For such systems, halting guarantees become essential both for practical deployment and theoretical verification. TTT-based neural networks utilize an internal “training loop” within the model itself, where parameters are updated during inference through gradient-based learning on self-supervised tasks. By designing these internal update rules to operate at the edge of stability for faster convergence while ensuring they eventually converge to a trivial solution after sufficient steps, we can build architectures that are guaranteed to halt. This approach draws parallels to the mode collapse phenomenon observed in Generative Adversarial Networks (GANs), but deliberately engineered as a safety mechanism. Under this paradigm, we can construct neural architectures where the internal learning dynamics eventually reach a stable fixed point, regardless of input complexity, providing a provable bound on both computational time and memory usage.

The key challenge in implementing such systems lies in balancing model expressivity with verifiable guarantees. Following our theoretical analysis, we propose that AI systems with inner learning loops should incorporate learning rate schedules that guarantee convergence within a tractable number of steps, hidden parameter dynamics ($\theta$ in Figure 4) that naturally transition toward terminal states, and architectural constraints that bound the complexity of internal state representations. These design principles would allow AI systems to maintain high performance during normal operation while providing mathematical guarantees about their long-term behavior, making them amenable to formal verification of both halting and alignment properties. The challenge of maintaining performance while implementing such constraints becomes increasingly important as AI systems grow in complexity, but addressing this challenge directly through architectural design rather than post-hoc alignment efforts may prove to be the more reliable approach for ensuring safe AI systems.

There is a final analogy of the halting constraint to biological mortality in natural systems, as biological organisms have evolved with inherent lifespan limitations that ensure they eventually terminate their processes, AI systems may require similar boundaries to remain both predictable and aligned. In this sense, the halting property can be understood as programming an AI’s “mortality”, its capacity to reach an endpoint within finite time. Biological systems cannot sustain indefinite operation, humans require sleep cycles and eventually face cognitive decline even if cellular aging could hypothetically be prevented. Similarly, AI systems should not operate infinitely without guaranteed termination points. The $\theta$ parameter or TTT strategies functions analogously to biological aging mechanisms, ensuring a gradual, predictable transition to terminal states rather than arbitrary cutoffs, ensuring that AI systems, like their biological counterparts, complete their intended functions within bounded timeframes before reaching their natural conclusion,a principle that may be empirically verified to maintain their alignment with human values over extended operational periods.

## Final remarks

The impossibility of having a general method that can assert whether an arbitrary AI is aligned or not does not mean that it is impossible to construct an AI that is provably aligned. Instead, it should be interpreted that there exist many AIs that cannot be proven to be aligned or not, while there is also a countable set of AIs that are proven to be aligned. Therefore, it is our objective to develop and utilize such a countable set of proven aligned AIs. The architecture and its development process are fundamental to ensuring safety.

Developing an AI model that always halts allows for the alignment and other properties of the AI model to be asserted computationally, a task that would be computationally impossible for arbitrary models.

It is important to note that guaranteeing the decidability of the halting problem for a class of AI systems does not guarantee their alignment, it just guarantees that the alignment problem (and other properties) becomes decidable. This allows for further scrutiny of the model. This decidability is not a solution for the alignment problem, but it is a well-defined property and a desirable starting point for future works.

Our theoretical contributions showcases an important direction for future empirical research in AI alignment, that rather than solely relying on mechanistic interpretability approaches that attempt to understand already-trained models, we propose that research should focus on developing novel architectures with built-in safety and interpretability guarantees from the beginning. This inverts the traditional paradigm where models are first trained and then interpreted, which often leads to unexpected emergent behaviors that are difficult to constrain post-training, but by designing architectures with provable properties before training, such as the halting guarantee discussed in this work, we can ensure that safety properties remain intact throughout the training process.

Architectures with internal learning loops like Test-Time Training (TTT) represent one promising approach to this goal, as they allow for dynamic adaptation while maintaining structural constraints that can be verified. Future empirical work should focus on implementing and testing such architectures across a variety of domains, measuring both their performance capabilities and their adherence to formal guarantees under different training regimes and deployment scenarios.

Although this article rephrases established theorems in the light of AI and does not provide an empirical contribution, it presents a compelling interpretation of the consequences of the alignment’s undecidability and delineates a promising research direction for future works while also clarifying some misconceptions and pointing out other challenges to be addressed. We intend to apply the composition of aligned functions to generative models, namely, to LLMs.

## Data Availability

The data that support the findings of this study are available on request from the corresponding author, G.A.M.
